# A dataset for distribution and characteristics of Holocene pyroclastic fall deposits along the Pacific coasts in western Hokkaido, Japan

**DOI:** 10.1016/j.dib.2020.106565

**Published:** 2020-11-23

**Authors:** Ryo Nakanishi, Juichiro Ashi, Satoshi Okamura

**Affiliations:** a Atmosphere and Ocean Research Institute, The University of Tokyo, Kashiwa 277-8564, Japan; b Graduate School of Frontier Sciences, The University of Tokyo, Kashiwa 277-8561, Japan; c Hokkaido University of Education, Sapporo 002-8501, Japan; d Hokkaido Soil Research Co-operation, Sapporo 003-0831, Japan

**Keywords:** Pyroclastic fall deposit, Tephra stratigraphy, Usu volcano, Tarumae volcano, Komagatake volcano, Hokkaido, Late Holocene

## Abstract

The tephra layers known with eruption ages play an important role in an investigation of tsunami history and archaeology in addition to volcanic history in Hokkaido, Japan. We investigated the event and tephra layers of the Late Holocene in the Pacific coast of western Hokkaido, where the stratigraphy of the Late Holocene has not been clarified. Surveys in coastal peatlands, mostly undisturbed deposits, have allowed for the discovery of thin tephra layers. The newly discovered tephra layers at the unexplored site were used to describe facies, observation under a polarization microscope, refractive index measurement of volcanic glasses, and chemical analysis, and correlated with the reported widespread tephras. We conducted wide-area field surveys and succeeded in revealing a wider distribution of tephra layers than previously known. The distribution of volcanic ash in the coastal area will contribute to the investigations of future volcanic and coastal hazards.

## Specifications Table

**Table:** 

Subject	Stratigraphy; Tephrochronology
Specific subject area	Volcanology; Geochemistry
Type of data	Tables and figures
How data were acquired	Geological fieldwork (sampling procedure) JEOL JXA 8900R (Electron Probe Micro Analyzer: EPMA). JEOL JSM-T330A, Link ISIS300 (Energy Dispersive X-ray Spectrometer: EDS) RIMS 2000 (Refractive Index Measuring System)
Data format	Raw
Parameters for data collection	The refractive index of volcanic glass was measured by dehydrating the samples so that the variation due to hydration was reduced. The chemical analysis of volcanic glass by EPMA and EDS was carried out by adjusting the current value and beam diameter to prevent the ionization of light elements.
Description of data collection	Layer thickness and stratigraphy were described from sampling core and coastal outcrops. The core samples were collected at several nearby sites and checked for variability.
Data source location	Samples were analyzed at the University of Tokyo, Kashiwa, Japan and Hokkaido University of Education, Sapporo, Japan. Sampling locations are listed in Fig. 1 and Tables 2.
Data accessibility	All the data sets are available with this article.

## Value of the Data

• Tephra distribution data are commonly used as chronological markers in Hokkaido.
• These data can contribute to reconstruct the magnitude and intensity of past explosive eruption in Hokkaido, Japan, and to model possible future eruptive scenarios for hazard assessment.
• The data can be used to constrain better the chronology of past coastal hazards (i.e., tsunami, storm) to assist archeological investigations for temporal evolution.

## 1 Data Description

**Fig. 1:**
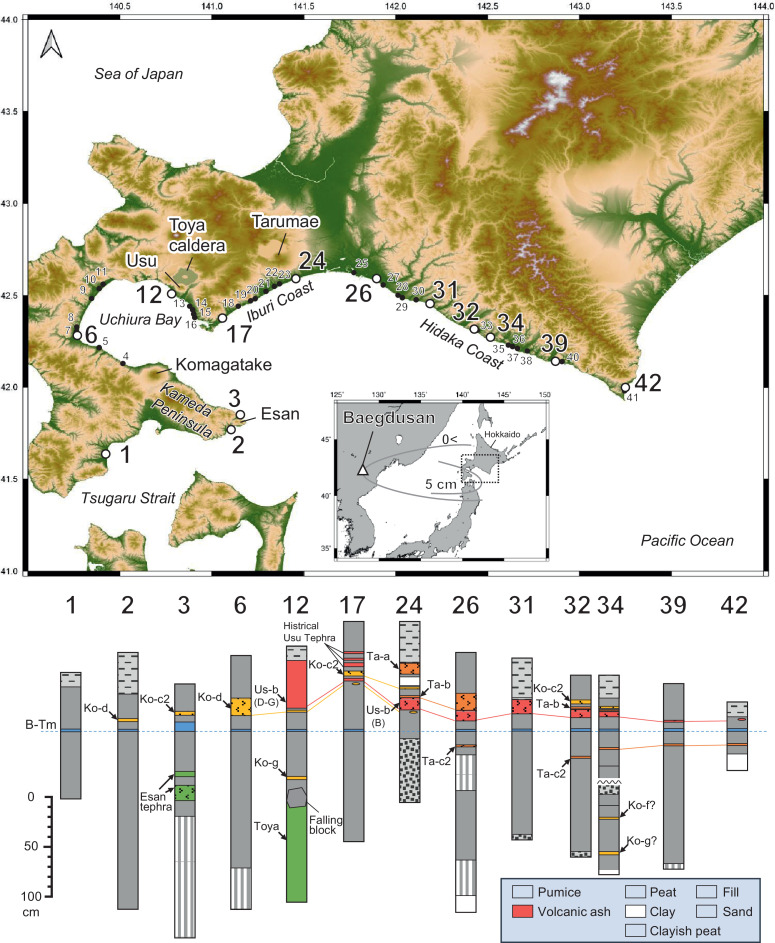
Upper: The topographic map (ASTER GDEM Version 3 [12]) of the survey site and the location of the stratigraphic columns. The inset shows the overall map of the study area and the location of Mt. Baegdusan. The solid black lines show the isopach of Baegdusan Tomakomai tephra (B-Tm). Lower: The typical example of stratigraphic columns of each region. The stratigraphic column of Sites 6 and 17 are based on Nakanishi and Okamura [6]. The stratigraphic column of Site 39 is based on Nakanishi et al. [7].

**Fig. 2:**
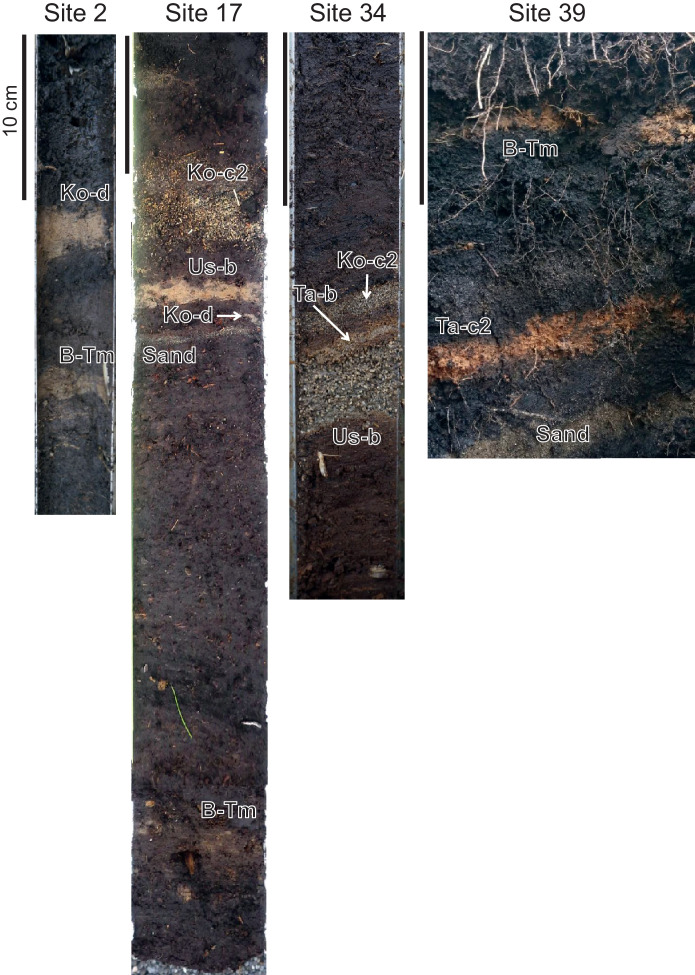
Photographs of cores and the outcrop at Sites 2, 17, 34, and 39.

**Fig. 3:**
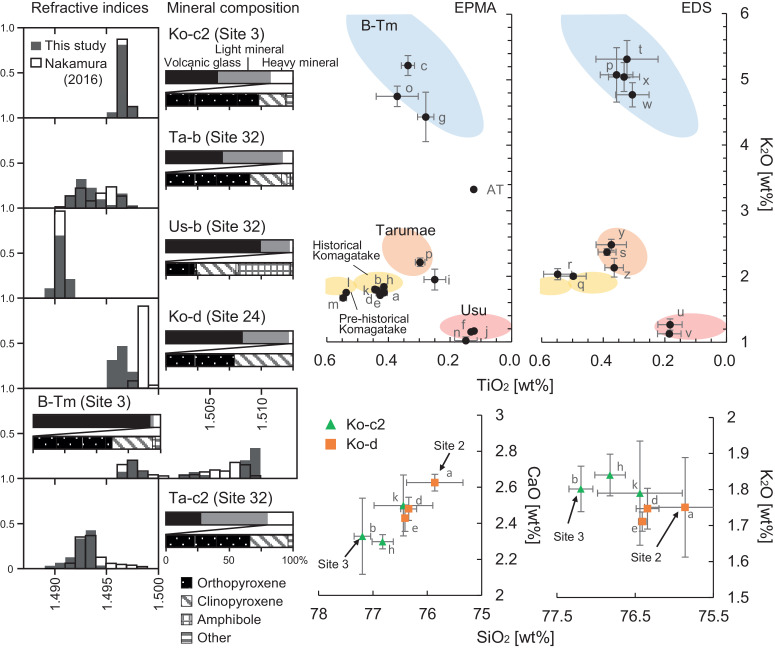
Left: Histograms of refractive index for volcanic glass and mineral composition. For comparison, the refractive index of the volcanic glass of Nakamura [1] is shown. Right: Variation diagrams for TiO_2_-K_2_O contents of glass shards by EPMA and EDS. Each symbol shows the mean and deviation in normalized wt%. Fields of referenced volcanic glass by Tokui [2], Aoki and Machida [5], Furukawa and Nanayama [3], and Nakamura [1] are color shaded. AT is a working standard sample analyzed in each analysis. Scatter plots for SiO_2_-K_2_O and SiO_2_-CaO contents of glass shards by EPMA to identify for historical Komagatake tephras.

Fig. 1 shows the study area and selected stratigraphic columns. Each volcanic ash layer was comprehensively correlated by comparing the layer facies (stratigraphic sequence, grain size, and coloration), mineral compositions, refractive index, and chemical composition of volcanic glass with those reported in Hokkaido [1], [2], [3]. The tephras widely distributed along the Pacific coast of western Hokkaido (Hidaka, Iburi, Uchiura bay, and Kameda Penisula) correspond to Komagatake c2 tephra (Ko-c2: AD1694), Tarumae b tephra (Ta-b: AD1667), Usu b tephra (Us-b: AD1663), Komagatake d tephra (Ko-d: AD1640), Baegdusan Tomakomai tephra (B-Tm: AD946), and Tarumae c2 tephra (Ta-c2: approximately BC400). The Us-b tephra is subdivided into units. The distribution is different between the Plinian eruption deposits (unit B) and the phreatomagmatic deposits (units A, C, E, F, and G: [1,4]). Fig. 2 shows photographs of representative cores and outcrops in each region. Table 1 shows the chemical composition of the confirmed tephra, and Fig. 3 shows the percentage of constituent minerals and the histogram of the refractive index. The scatter plots of K_2_O and TiO_2_, which are useful for the identification of volcanic ash [[1], [2], [3], 5], are presented as one of the premises on which we correlated unknown tephras of known age.

**Fig. 4:**
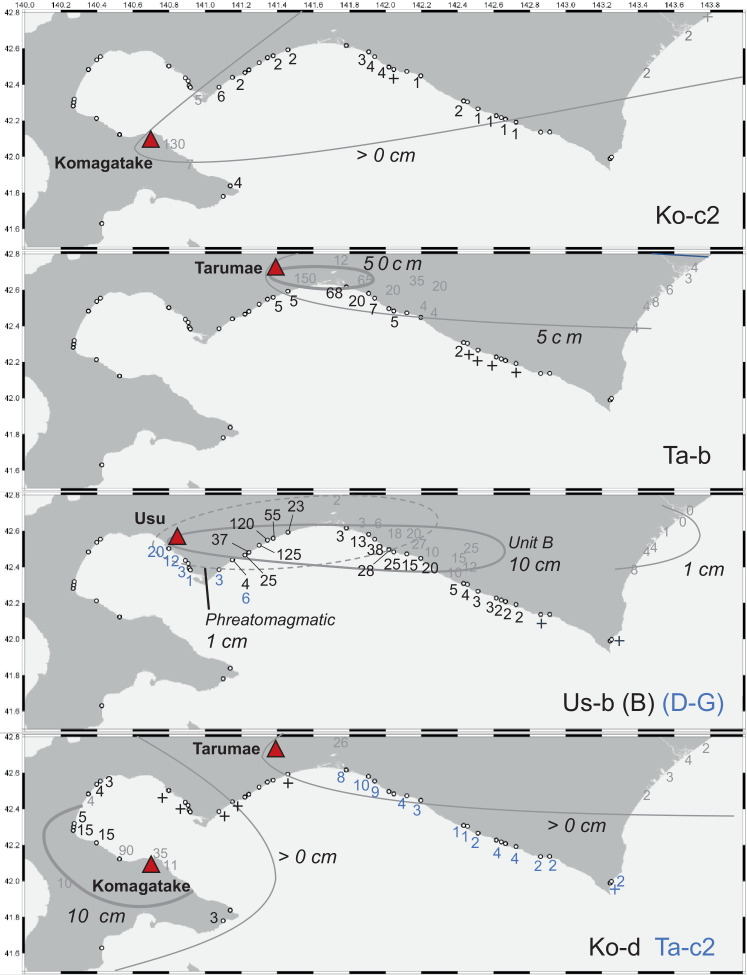
The open circles and black or blue numbers show the location of survey sites and thickness (cm) distribution of each widespread tephra revealed by this study, respectively. Cross marks show patchy tephra layers or thickness less than 1 cm. For comparison, the isopachs of each tephra from Nakamura [1] are shown as gray solid and dashed lines. Gray numbers and cross marks indicate the tephra thickness distribution reported by Tokui (1993), Furukawa and Nanayama [3], and Nakamura [1].

We describe the correlation of each tephra for each region where the combination of tephra layers is similar. In Kameda Peninsula and western Uchiura bay region, volcanic ash layers of Komagatake and B-Tm were identified. B-Tm was easily determined from the unique chemical composition (high potassium) and fine and good sorting grain at all sites. Komagatake tephras (Ko-d and Ko-c2) at Sites 2 and 3, which were difficult to identify from the stratigraphy and mineral compositions, were identified by the scattered plots of SiO_2_, K_2_O, and CaO [8]. From western Iburi to eastern Uchiura Bay, we identified B-Tm, Us-b: fine-grained phreatomagmatic units, Ko-c2 as coarse-grain and mafic minerals are widely observed. Ko-d tephra is occasionally found in patches below Us-b. Tokui [2] and Nakamura et al. [9] described in detail the stratigraphy of the eastern part of Iburi to the northern part of Hidaka; thus, we do not report here the details of the stratigraphy of this area. In the southern part of Hidaka, we identified Ta-c2, B-Tm, Us-b, Ta-b, and Ko-c2. Ta-c2 was found below B-Tm as dark orange and fine-grain volcanic ash. Peat layers rarely separate the three tephras deposited in the 17th century are rarely separated because of gaps of only a few years to a few decades. However, they can be distinguished based on the combination since unit B of Us-b is mainly pumice and Ta-b is fine-grain in contrast. The identification supported by the K_2_O-TiO_2_ diagrams is plotted in different areas due to the different source volcanoes of each tephra. Fig. 4 and Table 2 show the layer thickness distribution of tephras at each study site.

**Table 1:** Major element analyses of volcaniclastic glass. The result for each oxide is shown as the mean and deviation of normalized weight% and, N is a number of analyzed glass shards.

			Normalized average (%)											Standard deviation										

Sample ID	Site	Tephra name	SiO2	TiO2	Al2O3	FeO	MnO	MgO	CaO	Na2O	K2O	Raw total	N	SiO2	TiO2	Al2O3	FeO	MnO	MgO	CaO	Na2O	K2O	Raw total	Analysis equipment
AT		AT	78.65	0.12	11.90	1.26	0.03	0.13	1.10	3.47	3.33	93.0	10	0.07	0.00	0.03	0.03	0.00	0.00	0.01	0.06	0.02	0.66	EPMA
a	2	Ko-d	75.86	0.41	12.55	2.47	0.09	0.55	2.63	3.69	1.75	97.7	11	0.52	0.01	0.25	0.12	0.01	0.08	0.18	0.05	0.04	0.14	EPMA
b	3	Ko-c2	77.19	0.44	12.03	2.39	0.10	0.49	2.33	3.23	1.80	95.3	10	0.15	0.01	0.07	0.04	0.01	0.01	0.03	0.21	0.04	0.06	EPMA
c	3	B-Tm	70.06	0.34	13.49	4.69	0.13	0.10	0.90	5.07	5.22	96.5	12	0.98	0.02	0.52	0.11	0.01	0.01	0.11	0.22	0.15	0.24	EPMA
d	12	Ko-d	76.34	0.43	12.34	2.43	0.10	0.48	2.48	3.65	1.75	97.2	10	0.15	0.01	0.13	0.05	0.01	0.02	0.06	0.06	0.02	0.06	EPMA
e	24	Ko-d	76.41	0.43	12.25	2.48	0.09	0.52	2.43	3.69	1.71	97.3	11	0.05	0.01	0.03	0.02	0.01	0.01	0.03	0.07	0.03	0.03	EPMA
f	31	Us-b (B)	77.35	0.13	12.89	2.00	0.14	0.25	1.81	4.27	1.15	95.5	10	0.48	0.00	0.17	0.05	0.01	0.01	0.03	0.14	0.02	0.67	EPMA
g	31	B-Tm	73.03	0.28	11.88	4.07	0.10	0.09	0.72	5.39	4.43	95.8	10	0.84	0.03	0.66	0.27	0.01	0.04	0.19	0.21	0.38	1.08	EPMA
h	32	Ko-c2	76.82	0.41	12.16	2.30	0.09	0.45	2.30	3.63	1.84	97.1	10	0.20	0.01	0.10	0.04	0.00	0.01	0.09	0.04	0.03	0.06	EPMA
i	32	Ta-b	77.29	0.25	12.36	1.95	0.10	0.34	1.94	3.82	1.95	94.0	10	0.65	0.04	0.09	0.08	0.02	0.03	0.11	0.11	0.16	0.68	EPMA
j	32	Us-b (B)	77.17	0.12	12.96	1.92	0.16	0.25	1.85	4.41	1.17	94.9	10	0.71	0.00	0.12	0.01	0.01	0.00	0.02	0.10	0.01	0.93	EPMA
k	34	Ko-c2	76.44	0.44	12.41	2.48	0.10	0.49	2.50	3.35	1.79	97.0	10	0.54	0.01	0.26	0.08	0.01	0.02	0.17	0.17	0.04	0.14	EPMA
l	34	Ko-f?	75.05	0.54	12.56	2.83	0.11	0.62	2.63	3.91	1.75	97.4	11	0.09	0.01	0.05	0.03	0.01	0.01	0.04	0.07	0.02	0.04	EPMA
m	34	Ko-g?	74.32	0.55	13.03	2.95	0.11	0.66	3.03	3.69	1.67	97.5	11	0.27	0.01	0.22	0.06	0.00	0.01	0.12	0.05	0.04	0.09	EPMA
n	42	Us-b	75.31	0.15	14.43	1.67	0.12	0.26	2.54	4.50	1.02	95.8	10	0.72	0.04	0.52	0.16	0.01	0.06	0.24	0.13	0.06	0.21	EPMA
o	42	B-Tm	66.41	0.37	12.67	4.40	0.11	0.17	1.01	5.04	4.75	95.0	10	1.15	0.07	0.64	0.17	0.01	0.10	0.24	0.27	0.16	1.20	EPMA
p	42	Ta-c2	76.37	0.30	12.50	2.02	0.06	0.36	2.45	3.73	2.21	95.6	10	0.83	0.02	0.31	0.06	0.01	0.02	0.22	0.08	0.06	0.71	EPMA
q	2	B-Tm	71.77	0.36	14.13	4.15	0.06	0.10	1.12	3.15	5.07	100.2	10	1.42	0.05	0.72	0.42	0.04	0.04	0.36	0.32	0.41	0.42	EDS
r	9	Ko-d	75.81	0.50	13.47	2.38	0.15	0.49	2.47	2.74	2.00	100.4	9	0.14	0.04	0.16	0.08	0.06	0.03	0.10	0.13	0.03	0.08	EDS
s	17	Ko-d	75.98	0.55	12.52	2.16	0.10	0.55	2.10	3.13	2.04	98.5	10	0.38	0.05	0.10	0.10	0.05	0.06	0.07	0.19	0.08	0.12	EDS
t	32	Ta-c2	77.10	0.39	12.61	1.90	0.08	0.29	1.85	3.41	2.37	93.5	9	0.53	0.03	0.23	0.13	0.04	0.04	0.14	0.12	0.05	0.15	EDS
u	33	B-Tm	70.00	0.32	14.44	3.68	0.11	0.08	0.60	5.46	5.31	95.5	11	1.30	0.10	0.70	0.42	0.03	0.09	0.24	0.39	0.28	0.40	EDS
v	39	Us-b (B)	77.55	0.18	13.26	1.88	0.12	0.34	1.57	3.83	1.27	96.1	10	1.30	0.04	0.73	0.09	0.04	0.02	0.19	0.44	0.09	0.33	EDS
w	39	Us-b (B)	76.03	0.19	13.75	1.86	0.18	0.26	1.76	4.29	1.13	99.2	10	0.29	0.04	0.06	0.07	0.04	0.03	0.04	0.15	0.03	0.08	EDS
x	39	B-Tm	71.75	0.31	13.34	4.13	0.07	0.01	0.69	4.95	4.76	98.7	10	1.49	0.05	0.78	0.54	0.05	0.02	0.36	0.18	0.18	0.41	EDS
y	39	B-Tm	69.49	0.33	14.87	3.70	−0.03	0.07	0.63	5.90	5.04	96.7	10	1.14	0.05	0.78	0.55	0.03	0.02	0.13	0.66	0.22	0.40	EDS
z	39	Ta-c2	77.14	0.37	12.50	1.95	0.05	0.38	1.95	3.19	2.48	93.1	10	0.22	0.05	0.22	0.12	0.03	0.04	0.10	0.11	0.08	0.11	EDS
α	39	Ta-c2	76.49	0.36	13.08	1.63	0.08	0.35	2.35	3.52	2.13	95.5	11	0.59	0.03	0.34	0.13	0.05	0.05	0.23	0.14	0.14	0.19	EDS

**Table 2:** Latitude and longitude of the sampling site and thickness of the tephra layers. Bold letters indicate that pumice are the main component.

				Tephra layer thickness (cm)						Number of checked cores
Site	Place names	Latitude	Longitude	Ko-c2	Ta-b	Us-b B (F)	Ko-d	B-Tm	Ta-c2	
1	Shiriuchi	41.63015	140.42718					2–3		3
2	Hinohama	41.78051	141.10008				3–4	1–4		10
3	Todohokke	41.83841	141.13871	**4–8**				2–4		6
4	Washimoki	42.12321	140.52544				**90**			1
5	Nodaoi	42.21278	140.39756				**15**			2
6	Yakumo	42.28111	140.26867				**12–15**			10
7	Hanaura	42.30084	140.27149				**8–17**	2		15
8	Yamazaki	42.31947	140.27407				**3–6**	2		5
9	Nakanosawa	42.48374	140.35288				3–5	patch–1		17
10	Oshamanbe	42.53681	140.39934				1–3	1		4
11	Kyoritsu	42.5546	140.4197				2	1		1
12	Arutori	42.50259	140.79817			(16–50)	patch	2		5
13	Nakamareppu	42.4368	140.88948			(1)				1
14	Mareppu	42.41995	140.90502			(2–12)				3
15	Kogane	42.39475	140.91097			(3–4)	patch	1		5
16	Ishikawa	42.38394	140.91697			(1)				1
17	Wakayama	42.38551	141.07627	**2–8**		(1–4)	patch	1		102
18	Tomiura	42.44016	141.1509	**1–2**		**4**(6)	patch	1		11
19	Kojohama	42.46612	141.22053			**7–25**				5
20	Takeura	42.48162	141.24172			**23–52**				10
21	Hagino	42.52094	141.29922			**74–126**				7
22	Ishiyama	42.54912	141.34445			**104–122**				11
23	Shadai	42.55974	141.37612	**2–3**	**5**	**30–71**		1		33
24	Tarumai	42.59322	141.45847	**1–2**	**3–6**	**18–28**	patch			7
25	Atsuma	42.61677	141.7819		**68**	**3**		1	**6–8**	1
26	Taura	42.58127	141.90511		**20**	**13**		3	**10**	1
27	Shiomi	42.55456	141.93927	**2–4**	**7–21**	**38–55**		patch–1	**3–4**	9
28	Tomikawa	42.49727	142.01894	**4**		**15–51**				7
29	Monbetsu	42.48384	142.04563	patch	**2–7**	**20–35**		patch–3		9
30	Toyosato	42.47317	142.11813			**10–17**		patch–2		3
31	Kabari	42.44806	142.19574			**16–34**		patch–2	**patch–3**	21
32	Urawa	42.30936	142.43231	2	patch–2	**4–8**		patch–3	patch	20
33	Higashisizunai	42.30414	142.4541		patch	**4**		patch–2	patch–3	7
34	Harutachi	42.26573	142.51294	1	patch–1	**3**		1–2	2	7
35	Kerimai W	42.22778	142.61414		patch	**1–4**		1–2		4
36	Kerimai E	42.21743	142.64155			**1–2**		1	4	4
37	Hamahagifushi	42.20869	142.66531	1		**2**				1
38	Efue	42.19227	142.72283	patch–1	patch	**1–2**		2–3		4
39	Utoma	42.13652	142.85985			**patch–2**		patch–4	patch–4	30
40	Nishisamani	42.13736	142.91001					1	2	4
41	Tomabetsu	41.98969	143.24526					1–4	patch–3	7
42	Syoya	41.99826	143.25279			patch		2–5	patch	17

## 2 Experimental Design, Materials and Methods

### 2.1 Field survey

A total of 431 samples from cores and outcrops were obtained at the 42 sites along the Pacific coast by using a handy Geoslicer and a Peat Sampler (diameter of 7 cm) with lengths of either 0.6 m or 2.5 m. Photographs were taken of each core sample, and sedimentary facies (stratigraphic sequence, thickness, and presence of pumice) were described.

### 2.2 Sample preparation

To separate volcanic glass and rock-forming minerals from the clay, each sample was vibrated in an ultrasonic cleaning device and the clay components were removed with water elutriation. Samples were dried at 70 °C. The dried samples were divided into 0.063–0.125 phi and 0.125–0.25 phi using a sieve. Refractive index measurements and microscopic observations were conducted for samples of 0.063–0.125 phi and 0.125–0.25 phi, respectively. The samples measuring the glass refractive index were dehydrated by annealing at 400 °C for 12 h in an electric furnace [9].

### 2.3 Measurement of refractive index and mineral composition

At least 200 grains were counted under a polarization microscope to examine the mineral composition. The refractive index of volcanic glass shards was measured with a Refractive Index Measuring System (RIMS 2000: Kyoto Fission Track Co., Ltd.). This method is possible to measure with an overall accuracy of ±2 × 10^−4^ and a precision of ±1 × 10^−4^ [10].

### 2.4 Chemical analysis of volcanic glass

Chemical analysis of volcanic glass was performed using a JEOL JXA 8900R Electron Probe Micro Analyzer (EPMA) and a JEOL JSM-T330A (Link ISIS300) Energy Dispersive X-ray Spectrometer (EDS). EPMA operating conditions were 15 kV acceleration voltage, 7 nA beam current, beam scanned area of 10 μm, and counting time was 10–60 s at the peak position and 5–30 s at the background-position [11]. EDS operating conditions were 15 kV acceleration voltage, 1.1 nA specimen current, and the beam scanned an area of 3 μm. All analysis results were corrected using the oxide ZAF method. The AT tephra from Aira Caldera was used as an in-house standard to check any difference between the reference values [5]. The mean values of approximately 10 grain with a detection analysis values more than 90% by weight, and it was normalized to 100%.

## Declaration of Competing Interest

The authors declare that they have no known competing financial interests or personal relationships which have, or could be perceived to have, influenced the work reported in this article.

## References

[1] Nakamura Y. (2016). Stratigraphy, distribution, and petrographic properties of Holocene tephras in Hokkaido, northern Japan. Quat. Int..

[2] Tokui Y. (1989). Volcanic eruptions and their effects on human activity, in Hokkaido, Japan. Ochanomizu Chiri (Ochanomizu Geogr.).

[3] Furukawa R., Nanayama F. (2006). Holocene pyroclastic fall deposits along the Pacific coastal region of eastern Hokkaido. Bull. Volcanol. Soc. Jpn. (Kazan).

[4] Nakamura Y., Matsumoto A., Nakagawa M. (2005). Tephrochronological study of the AD1663 eruption of Usu volcano, western Hokkaido, Northern Japan. J. Tokyo Geogr. Soc..

[5] Aoki K., Machida H. (2006). Major element composition of volcanic glass shards in the late Quaternary widespread tephras in Japan e distinction of tephras using K_2_O-TiO_2_ diagrams. Bull. Geol. Surv. Japan (Chishitsu Chosa Kenkyu Houkoku).

[6] Nakanishi R., Okamura S. (2019). Tsunami deposits from the 1640 Hokkaido Komagatake eruption, north Japan: constraints on inundation heights and numerical simulation of volcanic debris avalanche-derived tsunami. J. Geol. Soc. Japan.

[7] Nakanishi R., Okamura S., Yokoyama Y., Miyairi Y., Sagayama T., Tsunami Holocene (2020). Storm and relative sea level records obtained from the Southern Hidaka Coast, Hokkaido, Japan. Quat. Sci. Rev..

[8] Takahashi R., Nakagawa M. (2013). Formation of a Compositionally Reverse Zoned Magma Chamber: petrology of the AD 1640 and 1694 Eruptions of Hokkaido-Komagatake Volcano, Japan. J. Petrol..

[9] Nakamura Y., Katayama Y., Hirakawa K. (2002). Hydration and refractive indices of Holocene tephra glass in Hokkaido, Northern Japan. J. Volcanol. Geotherm. Res..

[10] Danhara T., Yamashita Y., Iwano H., Kasuya M. (1992). An improved system for measuring refractive index using thermal immersion method. Quat. Int..

[11] Matsumoto A., Miyasaka M., Nakagawa M. (2015). Analysis of volcanic glass compositions using WDS-EPMA: examination of Na migration and proposal of analytical condition. Geophys. Bull. Hokkaido Univ..

[12] NASA/METI/AIST/Japan Spacesystems, and U.S./Japan ASTER Science Team (2019). ASTER Global Digital Elevation Model V003 [Data Set].

